# Feeding Period Restriction Alters the Expression of Peripheral Circadian Rhythm Genes without Changing Body Weight in Mice

**DOI:** 10.1371/journal.pone.0049993

**Published:** 2012-11-15

**Authors:** Hagoon Jang, Gung Lee, Jinuk Kong, Goun Choi, Yoon Jeong Park, Jae Bum Kim

**Affiliations:** 1 School of Biological Sciences, Institute of Molecular Biology and Genetics, Center for Adipose Tissue Remodeling, Seoul National University, Seoul, Korea; 2 Department of Biophysics and Chemical Biology, Seoul National University, Seoul, Korea; University of Hong Kong, China

## Abstract

Accumulating evidence suggests that the circadian clock is closely associated with metabolic regulation. However, whether an impaired circadian clock is a direct cause of metabolic dysregulation such as body weight gain is not clearly understood. In this study, we demonstrate that body weight gain in mice is not significantly changed by restricting feeding period to daytime or nighttime. The expression of peripheral circadian clock genes was altered by feeding period restriction, while the expression of light-regulated hypothalamic circadian clock genes was unaffected by either a normal chow diet (NCD) or a high-fat diet (HFD). In the liver, the expression pattern of circadian clock genes, including Bmal1, Clock, and Per2, was changed by different feeding period restrictions. Moreover, the expression of lipogenic genes, gluconeogenic genes, and fatty acid oxidation-related genes in the liver was also altered by feeding period restriction. Given that feeding period restriction does not affect body weight gain with a NCD or HFD, it is likely that the amount of food consumed might be a crucial factor in determining body weight. Collectively, these data suggest that feeding period restriction modulates the expression of peripheral circadian clock genes, which is uncoupled from light-sensitive hypothalamic circadian clock genes.

## Introduction

Various physiological and behavioral oscillations, such as sleep-wake cycles, body temperature, blood pressure, and hormone secretion, are associated with the circadian clock [1]. Circadian oscillation is composed of auto-regulatory negative feedback loops; Bmal1, Clock, Per, and Cry are key circadian transcription factors that produce rhythmic oscillations in a cell-autonomous manner. Bmal1 and Clock play key roles in inducing Per and Cry. Induced Per and Cry then form a transcriptional repressor complex to suppress Bmal1 and Clock, which eventually leads to negative feedback regulation. In addition, Bmal1 and Clock also increase the mRNA levels of Rev-erbα and RORα, which then compete for binding to the retinoic acid-related orphan receptor response elements (ROREs) and repress or activate the expression of Bmal1, respectively. This alternating promoter occupancy is due to the rhythmic expression of Rev-erbα [2]. Moreover, these auto-regulatory loops are modulated by various post-translational modifications such as phosphorylation, sumoylation, acetylation, and ubiquitination [3]–[5].

The circadian clock exists in the hypothalamic suprachiasmatic nucleus (SCN) and in peripheral tissues such as the liver and fat [1]. SCN circadian oscillation is primarily regulated by light, whereas peripheral circadian oscillation is affected by food intake along with hormones such as insulin and glucagon [2]. Emerging evidence suggests that the circadian clock is closely associated with whole-body energy homeostasis. For instance, Clock-defective mice exhibit obesity and hyperphagia with disrupted circadian oscillation [6]. High-fat diet (HFD)-fed wild-type (WT) mice become obese and show altered expression of circadian clock genes with metabolic dysregulation [7]. Further, liver-specific Bmal1 knockout mice lose their hepatic glucose homeostasis [8]. Moreover, key metabolic genes, such as PPARα, PPARγ, and AMPK, are involved in the regulation of circadian genes, and circadian clocks in turn modulate whole-body energy metabolism [4], [8], [9]. Under physiological conditions, the SCN and peripheral clocks are synchronized by the light/dark cycle and the feeding-fasting cycle. Nonetheless, it has been also demonstrated that disharmonious signaling by these two cues, the light/dark cycle and the feeding-fasting cycle, leads to the independent regulation of each circadian oscillation [10]. Although it appears that there is a close relationship between the circadian clock and metabolic regulation, the effects of unsynchronized SCN and peripheral-tissue circadian clocks on metabolic regulation are largely unknown. In addition, whether feeding period alteration would be a major determinant of body weight change even with an unchanged calorie intake is unclear.

In this study, we investigated whether feeding period restriction affects body weight gain in mice fed a NCD or HFD. We also analyzed the gene expression profiles of circadian clocks from hypothalamus (SCN) and peripheral tissue in mice with different feeding periods. Our data suggest that feeding period restriction does not influence body weight gain, whereas it differently regulates circadian oscillations in peripheral tissues but not in hypothalamus (SCN).

## Materials and Methods

### Animal Care and Experimental Protocol, Ethics Statements

**Figure 1:**
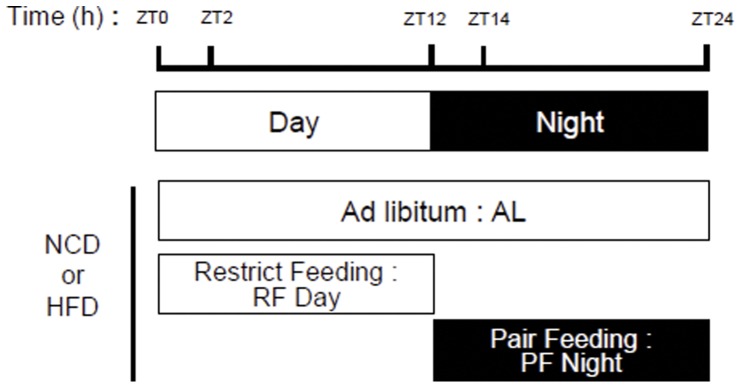
Feeding period restriction scheme for the three feeding groups in this study. The ad libitum (AL) group was freely exposed to food; the feeding restricted to day time (RF Day) group could access food only during daytime; and the pair-feeding at night (PF Night) group was given the same amount of food as the RF Day group but only at night. Mice were fed with a normal chow diet (NCD) or a high-fat diet (HFD). The light was turned on at ZT0 and turned off at ZT12. At ZT2 and ZT14, mice were sacrificed to prepare tissues and harvest blood samples.

The mice were maintained according to the guidelines of the Seoul National University Animal Experiment Ethics Committee. The protocol was approved by the committee on the Ethics of Animal Experiments of the Seoul National University (Permit Number: SNU-100316-4). Mice were euthanized if they met any early removal criteria (lethargy, hunched posture, or ruffled coat) to minimize suffering. Four-week-old male C57BL/6N mice were obtained from SAMTAKO BIO KOREA Co., Ltd. They were housed in individual cages for pair-feeding in 12 hr light/12 hr dark cycles. After a minimum 1-week stabilization period, ad libitum group mice (5 weeks old) were exposed to food freely, and the nighttime-fed mice were pair-fed with NCD and 60% HFD (Research Diets, Inc.) for 4 weeks to match the amount of food of the daytime group (Figure 1). Only those pair-fed mice (nighttime fed) with body weights similar to those of daytime-fed mice were subjected to the procedure from 5 weeks of age to 9 weeks of age. Body weight and food intake were measured daily at ZT0 (7 a.m.) and ZT12 (7 p.m.) during the experimental protocol. The average initial body weights in each group of mice were the same. After euthanized mice, dissected tissue specimens were frozen in liquid nitrogen and immediately stored at −80°C until analysis.

### Quantitative Real-time RT-PCR Analysis

Total RNA was isolated from liver and hypothalamus as described previously [11], and cDNA was synthesized using the M-MuLV reverse transcriptase kit (Fermentas, Glen Burnie, MD). The primers used for the real-time PCR analysis were produced by Bioneer (Korea). The primer sequences used for real-time PCR analysis are provided in Table S1.

### Biochemical Analysis

The levels of plasma cholesterol and triglycerides were measured using Infinity reagents (Thermo, Melbourne, Australia). Plasma glucose levels were measured with a freestyle blood glucose meter (Therasense; Uppsala, Sweden).

### Statistical Analysis

The results are shown as means ± SD. All statistical analyses were performed by Student *t* test or ANOVA (Student-Newman-Keuls comparison test as post hoc). *P*<0.05 was interpreted as being statistically significant.

## Results

### Feeding Period Restriction does not Change Body Weight Gain

To address the question of whether body weight gain is affected by feeding behavior and the accompanying changes in peripheral circadian clock genes, we investigated the effects of feeding period restriction on body weight. Feeding period restrictions were divided into the following three groups: 1) ad libitum, in which mice were freely exposed to food; 2) restriction feeding in daytime (RF Day), in which mice could access food only in daytime; and 3) pair-feeding in nighttime (PF Night), in which mice were given the same amount of food as the RF Day group but only at night (Figure 1). Because mice are nocturnal animals that consume more food at night than during the day, we measured the amount of food intake in the RF Day mice and gave the same amount of food to the nighttime-fed (PF Night) group. We designed the pair-feeding group to investigate the effect of feeding period variation with a constant calorie intake on body weight. Moreover, mice were fed with either a normal chow diet (NCD) or a high-fat diet (HFD) to test the effects of different nutrition sources on body weight gain upon feeding period restriction.

**Figure 2:**
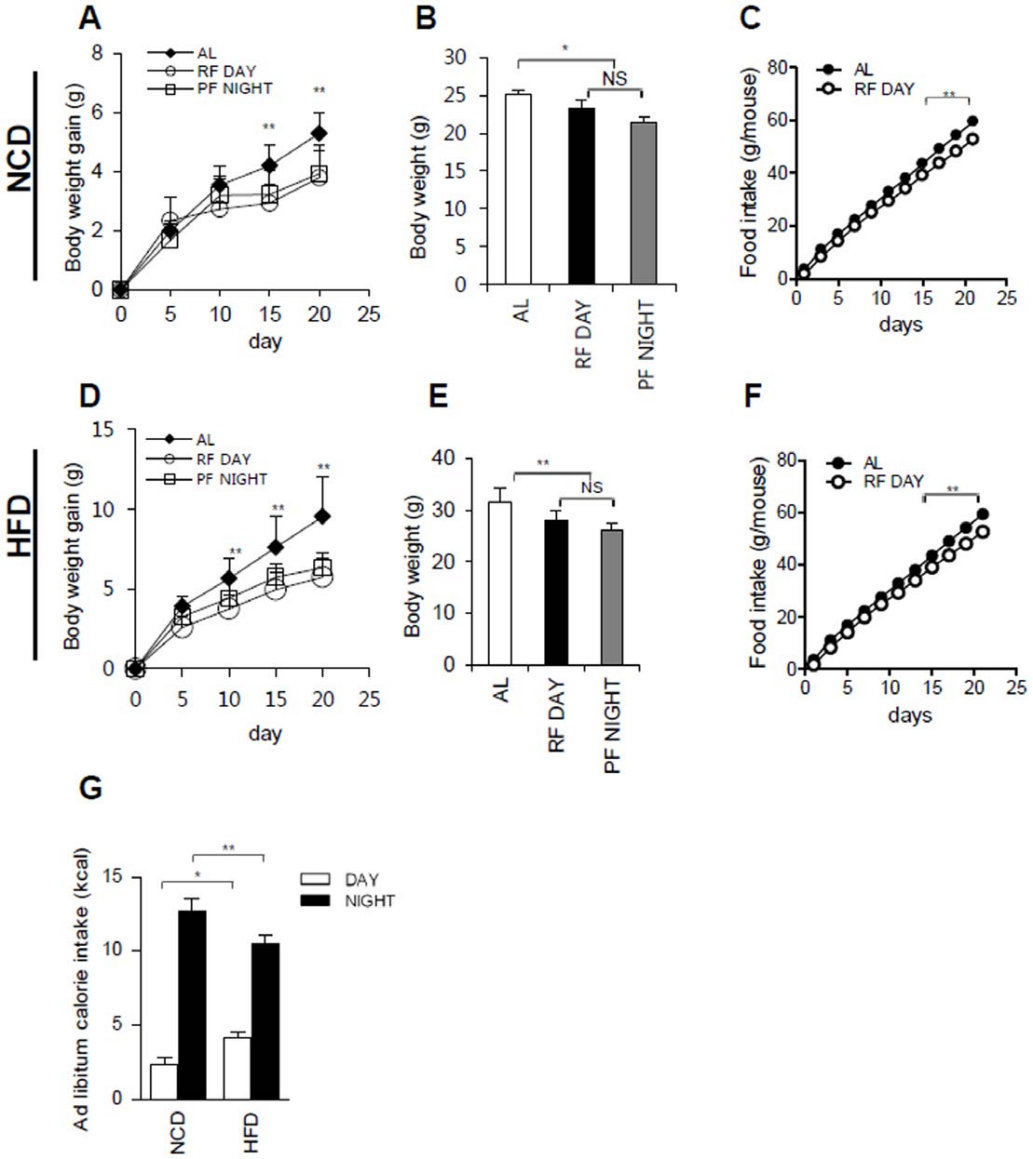
Feeding period restriction does not change body weight gain. ***A, B:*** Body weight gain or total body weight of AL, RF Day, and PF Night NCD-fed mice. ***C:*** Total food intake in NCD-fed AL and RF Day mice. ***D, E:*** Body weight gain or total body weight of AL, RF Day, and PF Night HFD-fed mice. ***F:*** Total food intake in HFD-fed AL and RF Day mice. ***G:*** Ad libitum calorie intake during day and night with NCD and HFD feeding. Each bar represents mean ± SD of each group of mice (n = 6), *P<0.05, **P<0.01.

Interestingly, we observed that the RF Day and PF Night groups displayed a similar pattern of body weight gain, regardless of the diet (NCD or HFD) (Figure 2A, 2B, 2D, and 2E). Compared to the RF Day and PF Night restricted feeding groups, the body weight gain of the ad libitum group significantly increased with either NCD or HFD (Figure 2A, 2B, 2D, and 2E). When cumulative food intakes were measured, the ad libitum group gradually consumed more food than the RF Day and PF Night groups (Figure 2C and 2F). Recently, it has been reported that the circadian clock is disrupted by HFD [7]. Thus, we examined the food intake patterns of the ad libitum group during the day (ZT0-ZT12) and night (ZT12-ZT24) time periods. As expected, mice ate more food during the night than during the day (Figure 2G). Interestingly, it seemed that the HFD-fed mice consumed slightly, but significantly, more food during the day than NCD-fed mice (Figure 2G). These data suggest that the amount of food eaten, rather than the feeding period, is a major determinant of body weight gain, even with different nutrition sources such as NCD and HFD.

### Daytime Feeding Changes the Expression of Circadian Clock Genes in the Liver but not in the Hypothalamus

**Figure 3:**
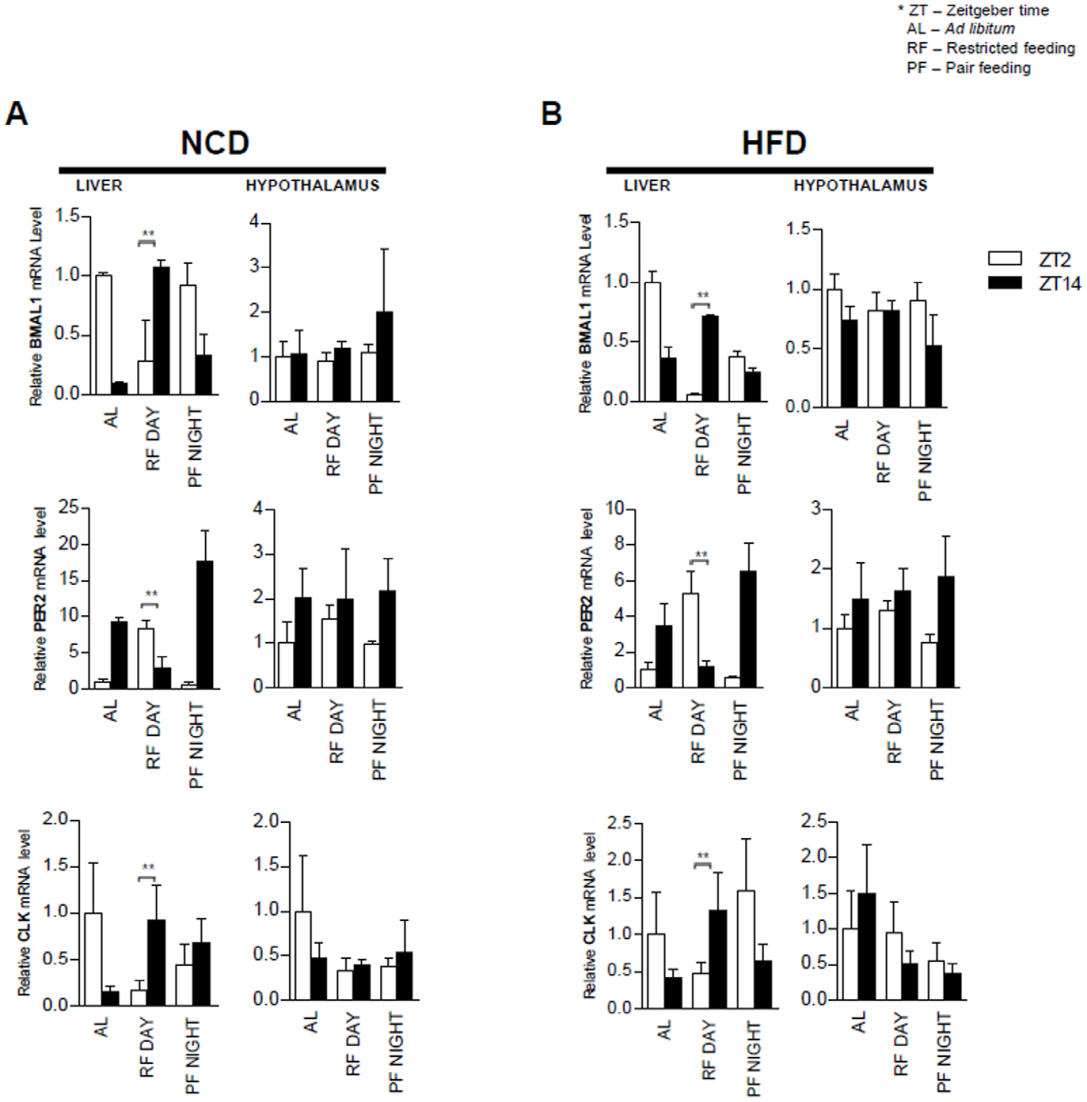
Daytime feeding changes expression of circadian clock genes in the liver but not in the hypothalamus. ***A:*** Hepatic and hypothalamic Bmal1, Per2, and Clock gene expression profiles in AL, RF Day, and PF Night NCD-fed mice at ZT2 and ZT14. ***B:*** Hepatic and hypothalamic Bmal1, Per2, and Clock gene expression profiles in AL, RF Day, and PF Night HFD-fed mice at ZT2 and ZT14. All RNA samples are normalized to TBP mRNA. Each bar represents mean ± SD of each group of mice (n = 3), *P<0.05, **P<0.01.

We next investigated the expression of circadian clock genes upon feeding period restriction. To examine the expression profiles of circadian clock genes, mice were sacrificed at ZT2 and ZT14, which may reflect different circadian clock gene expression patterns in a 12∶12-hour light/dark cycle. Total RNA was isolated from liver and hypothalamus, representing peripheral clock and central clock tissues, respectively, and analyzed by qRT-PCR. As shown in Figure 3, the expression patterns of circadian clock genes, such as Bmal1, Per2, and Clock, were altered in the liver but not in the hypothalamus by feeding period restrictions with either NCD or HFD. Overall, the ad libitum group and PF Night group showed the most similar expression patterns of circadian clock genes in the liver, which would result from the nocturnal behavior of ad libitum mice that took most of their food during the night. In contrast, the RF Day group revealed an expression pattern of circadian genes in the liver that was distinct from the ad libitum and PF Night groups. Unlike expression in the liver, the expression patterns of hypothalamic circadian genes were not changed by feeding period restrictions with either NCD or HFD (Figure 3A and 3B). These data strongly indicate that feeding period restriction influences the expression of circadian clock genes in peripheral tissues, probably by modulating nutritional hormones, such as insulin or glucagon that may not affect the expression of circadian clock genes in the hypothalamus.

### Feeding Period Restriction Alters the Expression of Metabolic Genes and Plasma Metabolites

**Figure 4:**
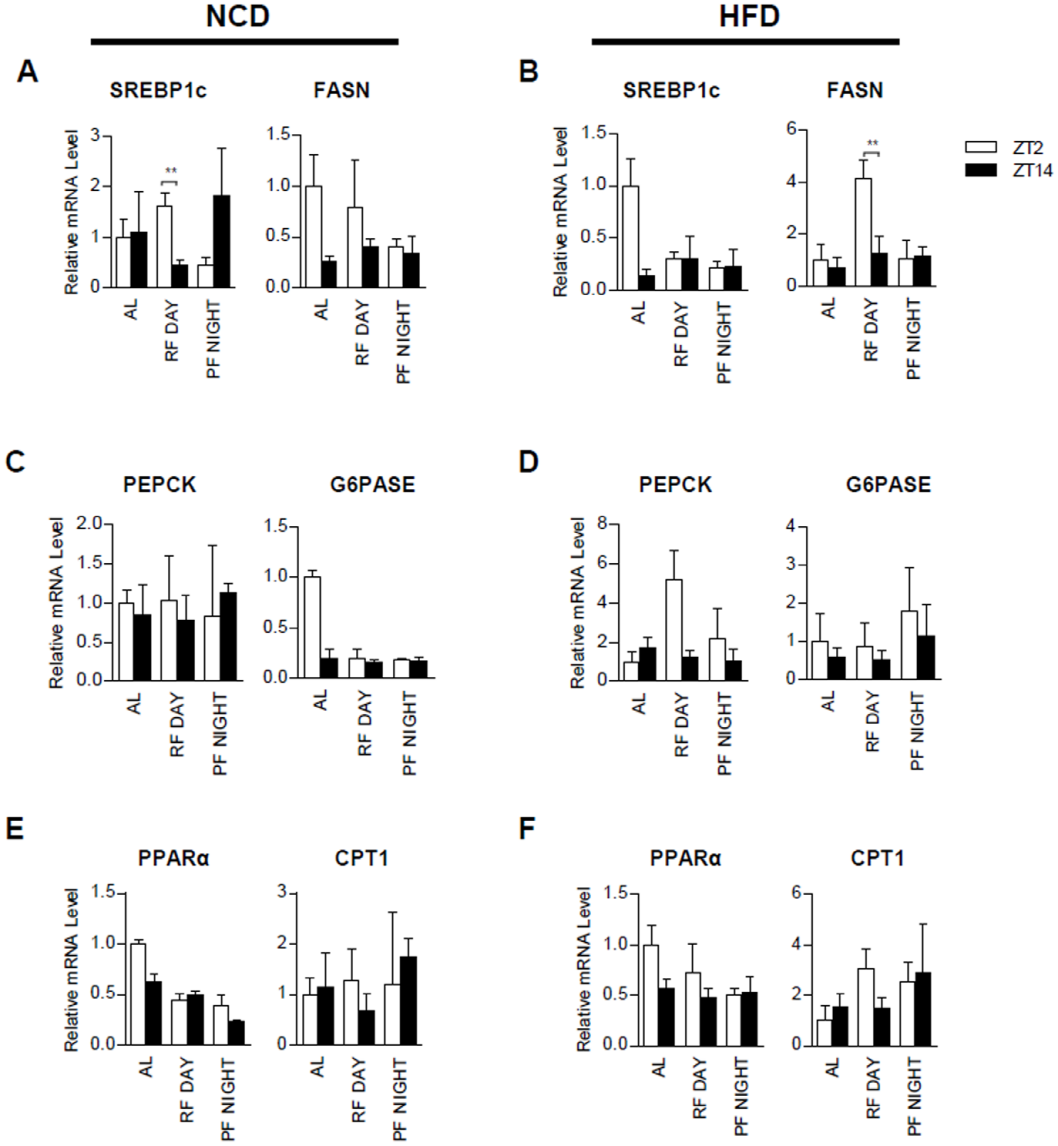
Feeding period restriction changes the expression of metabolic genes. ***A:*** Expression profiles of lipogenic genes such as SREBP1c and FASN in AL, RF Day, and PF Night NCD-fed mice at ZT2 and ZT14. ***B:*** Expression profiles of lipogenic genes such as SREBP1c and FASN in AL, RF Day, and PF Night HFD-fed mice at ZT2 and ZT14. ***C:*** Expression profiles of the gluconeogenic genes PEPCK and G6Pase in AL, RF Day, and PF Night NCD-fed mice at ZT2 and ZT14. ***D:*** Expression profiles of the gluconeogenic genes PEPCK and G6Pase in AL, RF Day, and PF Night HFD-fed mice at ZT2 and ZT14. ***E:*** Expression profiles of the fatty acid oxidation genes PPARα and CPT1 in AL, RF Day, and PF Night NCD-fed mice at ZT2 and ZT14. ***F:*** Expression profiles of the fatty acid oxidation genes PPARα and CPT1 in AL, RF Day, and PF Night HFD-fed mice at ZT2 and ZT14. All RNA samples are normalized to TBP mRNA. Each bar represents mean ± SD of each group of mice (n = 3), *P<0.05, **P<0.01.

It is well known that feeding is an important factor for regulating circadian clock genes and hepatic lipid and glucose metabolism [7], [12]. Given that the RF Day group displayed changed expression patterns of hepatic circadian clock genes, we examined whether RF Day might also influence the expression of metabolic genes in the liver. We analyzed hepatic gene expression using qRT-PCR from NCD (Figure 4A, 4C, and 4E) or HFD (Figure 4B, 4D, and 4F) fed mice at ZT2 and ZT14. In order to uncover key metabolic changes, we investigated the expression of several lipid and carbohydrate metabolism genes, including SREBP1c, FASN, PEPCK, G6Pase, PPARα, and CPT1. Because SREBP1c is a master transcription factor for lipogenesis, it regulates the expression of FASN upon nutritional and hormonal changes [13]–[17]. With NCD feeding, the expression of SREBP1c mRNA was greatly suppressed in RF Day mice at ZT14, while it was upregulated at ZT14 in PF Night mice (Figure 4A). Despite this pattern, FASN did not significantly change between ZT2 and ZT14 in RF Day or PF Night, implying that it may take several hours to detect SREBP1c target gene expression in vivo. Upon HFD feeding, FASN was increased in RF Day at ZT2, but it was not altered in PF Night (Figure 4B). Conversely, the expression of gluconeogenic genes, such as PEPCK and G6Pase, which are well known fasting-induced genes, did not change between ZT2 and ZT14 in either RF Day or PF Night mice with NCD (Figure 4C). The mRNA levels of fatty acid oxidation genes, such as PPARα and CPT1, were not significantly different between the RF Day and PF Night groups (Figure 4E). These data suggest that while the expression levels of several metabolic genes may be partially altered by feeding period restriction, they are not altered as much as hepatic circadian clock genes.

Because feeding period restriction altered the expression of a subset of metabolic genes in the liver without causing body weight gain, we examined serum metabolites that are relevant to the whole-body energy state in different feeding period restriction groups. Although it has been reported that serum metabolite levels vary throughout the feeding-fasting cycle [18], our experimental windows, limited to ZT2 and ZT14, did not sensitively reflect the whole-body energy state. When we measured serum glucose, triglyceride (TG), and cholesterol levels at ZT2 and ZT14, we failed to observe distinct patterns of those metabolites. Nonetheless, in the PF Night group, the levels of serum glucose, TG, and cholesterol were higher at ZT2 than at ZT14 under NCD, implying that there might be a correlation between peripheral circadian oscillation and serum metabolites (Figure S1A–C). In contrast, HFD-challenged animals exhibited disrupted serum metabolite profiles, as previously reported (data not shown) [19]. These data indicate that feeding period restriction may modulate the profiles of several serum metabolites, without changes in body weight gain, while concomitantly altering the expression of circadian clock genes and metabolic genes.

## Discussion

Obesity is characterized by increased adipose tissue and lipid metabolism, which are caused by chronic excesses of energy intake over energy expenditure. Very recent reports have suggested that feeding period restriction may be an important factor influencing body weight gain and leading to obesity [19], [20]. Since Arble et al. reported that daytime HFD-fed mice were more obese than nighttime HFD-fed mice with identical calorie intake and activity [20], it has been proposed that shifting feeding behavior from night to day is an important factor contributing to the regulation of energy metabolism. Accordingly, it has been reported that night eating syndrome in humans might lead to weight gain as a result of excess calorie consumption at night [21]–[23]. On the other hand, several studies have found no correlation between night eating syndrome and body weight gain in humans [24]–[26]. Nonetheless, it has not been clearly understood whether the dysregulation of expression in any circadian clock gene is essential for altering body weight gain.

In the present work, we investigated the relationship between body weight gain and feeding period restriction by providing the same amount of food either during the day or during the night. We observed that feeding period restriction altered the expression patterns of peripheral circadian clock genes, probably via nutrition-sensitive hormones. However, the expression of central circadian clock genes in the hypothalamus was not modulated by feeding restriction in either NCD or HFD. These data imply that the central and peripheral circadian clocks are independently regulated, at least at the transcriptional level, upon feeding period restriction [10]. Furthermore, the hepatic expression of metabolic genes involved in lipogenesis, gluconeogenesis, and fatty acid oxidation was modulated upon feeding period restriction. Similarly, the levels of serum glucose, TG, and cholesterol were changed by feeding period restriction. Despite these changes in peripheral metabolic gene expression, there were no significant body weight differences between the RF Day and PF Night groups with NCD or HFD, indicating that the change in peripheral circadian clock gene expression may not be a key factor influencing body weight. Instead of feeding behavior, the amount of energy intake appears to be a crucial factor in determining body weight and may be closely linked with central circadian clock genes.

It has been reported that HFD challenge disrupts behavioral and physiological circadian rhythms. For instance, HFD leads to alterations in the periods of the locomotor activity and canonical circadian clock genes [9]. In this study, we observed that HFD-fed mice exhibited distinct expression patterns of a subset of genes in the liver, including circadian clock genes and metabolic genes, compared with NCD-fed mice. In addition, the ad libitum group exhibited different food consumption patterns that depended upon the nutrition source. HFD-challenged mice consumed more food during the day than NCD-fed mice, implying that food intake control is dysregulated in the HFD-fed group. As the fasting period of the HFD-fed group might be shorter than that of NCD-fed group during ad libitum feeding [7], the NCD-fed group might gain less body weight than the HFD-fed group because of the activation of fasting-induced signals, such as SIRT1 and AMPK, which mediate energy expenditure to burn extra energy [7], [27]. However, we cannot rule out the possibility that physical activity or body temperature may help compensate for body weight gain; this possibility will be investigated in a further study.

Disruption of circadian clock genes is triggered by various stimuli such as changes in the light/dark cycle and food consumption. Interference of the central circadian clock by light/dark cycle modulation affects body weight gain [28]. For instance, a 10∶10-hour light/dark (LD) cycle leads to body weight gain compared with a 12∶12-hour LD cycle, and a 10∶10-hour LD cycle regime increases metabolic parameters, such as plasma leptin, insulin, and glucose [28]. Moreover, circadian clock-defective mice, such as clock gene mutant mice and HFD-fed mice, show irregular food intake patterns that are accompanied by obesity due to increased food intake [6], [10]. In contrast, Bmal1-deficient mice display an obese phenotype during 5 weeks of HFD, whereas they are no longer obese after 15 weeks of HFD [29]. However, liver-specific Bmal1 knockout mice show more body weight gain than WT mice [12].

Here, we have shown that feeding period restriction selectively alters the expression patterns of peripheral circadian clock genes without inducing body weight gain. Concomitantly, feeding-restricted mice show altered metabolic gene expression levels and plasma profiles. Therefore, it is plausible to speculate that the central circadian clock might play a key role in regulating body weight by modulating energy intake and expenditure. Consistent with this idea, it appears that total calorie intake is a major factor in body weight. In contrast, peripheral circadian clock genes sensitively respond to metabolic alterations, likely because of changes in nutrients and hormones. The exact molecular mechanism by which the central circadian clock influences body weight gain remains to be elucidated. In conclusion, our data suggest that feeding period restriction selectively modulates peripheral circadian clocks and glucose and lipid metabolism without significantly changing body weight.

## Supporting Information

Figure S1 **Feeding period restriction changes plasma metabolites.** ***A:*** Serum glucose levels in AL, RF Day, and PF Night NCD-fed mice at ZT2 and ZT14. ***B:*** Serum TG levels in AL, RF Day, and PF Night NCD-fed mice at ZT2 and ZT14. ***C:*** Serum cholesterol levels in AL, RF Day, and PF Night NCD-fed mice at ZT2 and ZT14. Each bar represents mean ± SD of each group of mice (n = 3), *P<0.05, **P<0.01.
(PDF)

Table S1 **Sequences of primers for real-time PCR.**
(PDF)
