# Supplementary figures and images for: Feeding Period Restriction Alters the Expression of Peripheral Circadian Rhythm Genes without Changing Body Weight in Mice
Source: PLoS One. 2012 Nov 15;7(11):e49993. doi: 10.1371/journal.pone.0049993 (PMC3499481; doi:10.1371/journal.pone.0049993)

Figure S1

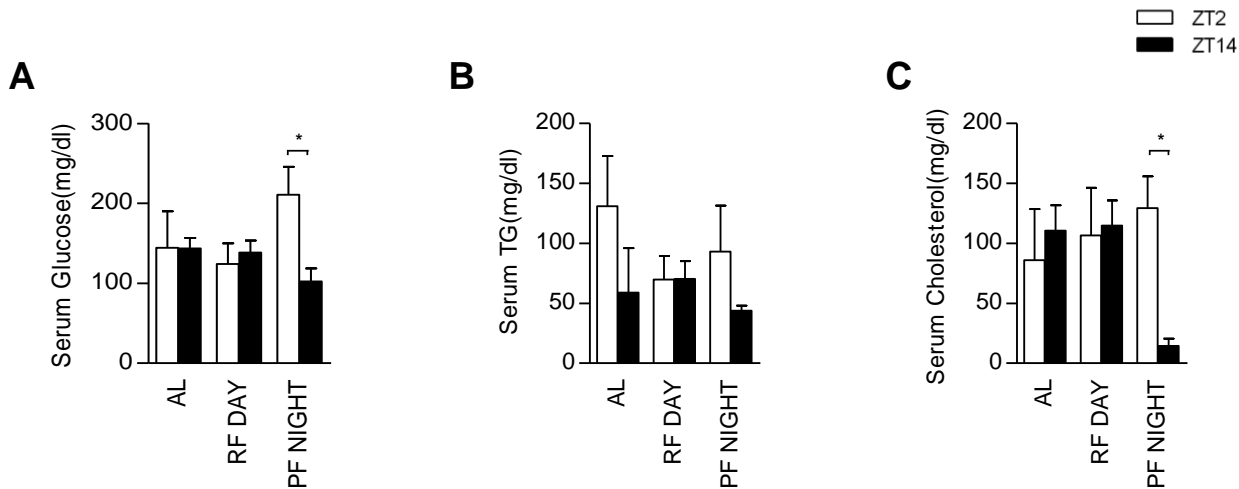

Supplement: Figure S1 — Feeding period restriction changes plasma metabolites. A: Serum glucose levels in AL, RF Day, and PF Night NCD-fed mice at ZT2 and ZT14. B: Serum TG levels in AL, RF Day, and PF Night NCD-fed mice at ZT2 and ZT14. C: Serum cholesterol levels in AL, RF Day, and PF Night NCD-fed mice at ZT2 and ZT14. Each bar represents mean ± SD of each group of mice (n = 3), *P<0.05, **P<0.01. (PDF) [file pone.0049993.s001.pdf]
