# Supplementary material for: Feeding Period Restriction Alters the Expression of Peripheral Circadian Rhythm Genes without Changing Body Weight in Mice
Source: PLoS One. 2012 Nov 15;7(11):e49993. doi: 10.1371/journal.pone.0049993 (PMC3499481; doi:10.1371/journal.pone.0049993)
Supplement: Table S1 — Sequences of primers for real-time PCR. (PDF) [file pone.0049993.s002.pdf]

Table S1.
Sequences of Primers for Real-Time PCR

Gene	Primer Sequence
Clock	5’-TTGCGTCTGTGGGTGTTG-3’<br>5’-TGCTTTGTCCTTGTCATCTTCT-3’
Bmal1	5’-AACCTTCCCGCAGCTAACAG-3’<br>5’-AGTCCTCTTTGGGCCACCTT-3’
Per2	5’-TGTGCGATGATGATTCGTGA-3’<br>5’-GGTGAAGGTACGTTTGGTTTGC-3’
TBP	5’-GGGAGAATCATGGACCAGAA-3’<br>5’-CCGTAAGGCATCATTGGACT-3’
Srebp1c	5’-GGAGCCATGGATTGCACATT-3’<br>5’-CAGGAAGGCTTCCAGAGAGG-3’
Fasn	5’-GCTGCGGAAACTTCAGGAAAT-3’<br>5’-AGAGACGTGTCACTCCTGGACTT-3’
G6pase	5’-ACACCGACTACTACAGCAACAG-3’<br>5’-CCTCGAAAGATAGCAAGAGTAG-3’
Pepck	5’-AAAAGCCTTTGGTCAACAAC-3’<br>5’-AAACTTCATCCAGGCAATGT-3’
Cpt1	5’-ACTCCTGGAAGAAGAAGTTCAT-3’<br>5’-AGTATCTTTGACAGCTGGGAC-3’
Pparα	5’-ATGCCAGTACTGCCGTTTTC-3’<br>5’-GGCCTTGACCTTGTTCATGT-3’
